# Distribution of and Relationships between Epidemiological and Clinicopathological Parameters in Canine Leishmaniosis: A Retrospective Study of 15 Years (2009–2023)

**DOI:** 10.3390/pathogens13080635

**Published:** 2024-07-29

**Authors:** Ricardo Lopes, Andreia Garcês, Augusto Silva, Paula Brilhante-Simões, Ângela Martins, Elsa Leclerc Duarte, Ana Cláudia Coelho, Luís Cardoso

**Affiliations:** 1 Department of Veterinary Sciences, University of Trás-os-Montes e Alto Douro (UTAD), 5000-801 Vila Real, Portugal; lopes.rmv@gmail.com (R.L.); accoelho@utad.pt (A.C.C.); 2 Department of Veterinary and Animal Sciences, University Institute of Health Sciences (IUCS), CESPU, 4585-116 Gandra, Portugal; paulabrilhante@inno.pt; 3 Wildlife Rehabilitation Centre (CRAS), Veterinary Teaching Hospital, University of Trás-os-Montes e Alto Douro (UTAD), 5000-801 Vila Real, Portugal; andreiamvg@gmail.com; 4 Animal and Veterinary Research Centre (CECAV), Associate Laboratory for Animal and Veterinary Sciences (AL4AnimalS), University of Trás-os-Montes e Alto Douro (UTAD), 5000-801 Vila Real, Portugal; angela@utad.pt; 5 INNO Veterinary Laboratories, R. Cândido de Sousa 15, 4710-300 Braga, Portugal; augustosilva@inno.pt; 6 Department of Veterinary Medicine, School of Science and Technology, University of Évora, Polo da Mitra, Apartado 94, 7002-554 Évora, Portugal; emld@uevora.pt; 7 Mediterranean Institute for Agriculture, Environment and Development (MED) & Global Change and Sustainability Institute (CHANGE), University of Évora, Polo da Mitra, Apartado 94, 7002-554 Évora, Portugal

**Keywords:** clinical pathology, dogs, enzyme-linked immunosorbent assay, *Leishmania infantum*, One Health, Planetary Health, Portugal, Stockholm Paradigm, vector-borne diseases, zoonosis

## Abstract

Leishmaniosis is a vector-borne disease caused by protozoan parasites of the genus *Leishmania*, which are zoonotic and have an important impact on animal and public health globally. Between 2009 and 2023, blood samples from domestic dogs with clinical suspicion of leishmaniosis were received from 286 veterinary medical centres throughout mainland Portugal. An enzyme-linked immunosorbent assay (ELISA) was utilised to detect antibodies against *Leishmania infantum* antigens. Additionally, a complete blood count and tests for total proteins, urea, creatinine and alanine aminotransferase, as well as protein electrophoresis, were also performed. No significant relationship between sex and breed was observed. The age distribution was bimodal, with the highest prevalence of disease occurring at 2–5 years of age and a secondary peak occurring at 6 years or over (*p* < 0.001). No statistical correlation was observed between creatinine and urea across the ELISA serological groups. In contrast, both the gamma globulin levels (r = 0.45; *p* < 0.001) and the albumin/globulin ratio (r = −0.36; *p* < 0.001) exhibited moderate correlations with the ELISA. These findings support recent seroprevalence studies in dogs, with some geographical areas in Northern Portugal exhibiting the highest values, which may be the result of geographical shifts in parasite circulation due to climate change.

## 1. Introduction

Canine leishmaniosis (CanL) is a vector-borne disease caused by protozoan parasites of the genus *Leishmania*, considerably impacting both human and veterinary public health globally [1,2]. The disease is predominantly observed in the Mediterranean region, South and Central America and selected Asian territories [3,4,5]. In Portugal, CanL is present throughout the mainland, with a higher prevalence in the interior centre and south of Portugal according to the latest studies [6,7]. The complex epidemiological profile is sculpted by an array of factors, including environmental conditions, host–pathogen interactions and vector phenology [8,9,10]. The vectors (phlebotomine sand flies) are influenced by diverse climatic conditions [11,12,13]. This dictates the prevalence of the disease and its spatial distribution, highlighting the complex interplay between vector availability, host genetic susceptibility and environmental exposure [14,15,16,17]. Clinically, CanL manifests with a broad spectrum of clinical signs, ranging from cutaneous lesions and renal dysfunction to systemic involvement, thus complicating its diagnosis and therapeutic management [18]. Previous studies have explored the correlation between clinicopathological parameters and other epidemiological factors, such as age, demonstrating that young dogs develop systemic, renal and haematological abnormalities less frequently compared to older dogs, while dermatologic signs are more common in young and adult dogs. The main clinicopathological abnormalities include hyperglobulinemia, hypoalbuminemia, mild to moderate non-regenerative anaemia and mild to severe proteinuria [19]. The heterogeneity in clinical presentation is intricately associated with the virulence of the infecting *Leishmania* strain, the immunological status of the host and the potential for co-infections with other vector-borne pathogens, which may further complicate the clinical scenario [20,21,22,23].

Moreover, the (subclinical) carriage and the reservoir potential of domestic and wild canines underscore the complex epidemiology of the disease, posing challenges to control and prevention strategies [24]. The diagnosis of CanL is complex, and multiple parameters need to be considered [8]. Serological procedures are widely used in diagnosing canine leishmaniosis due to their high sensitivity and specificity [18,25,26,27], ability to detect subclinical infections [28,29,30] and practicality in terms of non-invasive sample collection [18,29,30,31]. These tests are essential for early diagnosis and effective treatment and control of the disease [28,29]. Combining serological methods with other diagnostic techniques further enhances their accuracy and reliability [6,31,32].

The expansion of CanL’s geographical distribution in recent years has been notably accelerated by climate change, augmented animal mobility across international borders and urbanisation trends, all contributing to the enlargement of vector habitats and subsequent alterations in disease transmission dynamics [1,13,33,34]. This evolving scenario underscores the need for an integrated approach to health, aligning with the Stockholm Paradigm within the Planetary Health concept, which emphasises the interdependence of human, animal and environmental health [13,35,36,37,38,39]. By situating CanL within this paradigm, we aim to contribute to a more integrated understanding of zoonotic diseases and reinforce the necessity of a comprehensive strategy to mitigate the impact of infectious diseases on a global scale [40,41].

This paper aims to present an update to the distribution patterns and the interplay among the epidemiological and clinicopathological parameters of CanL in the Portuguese canine population during a period of 15 years (2009–2023) and determine possible associations and correlations between different variables related to this disease.

## 2. Materials and Methods

### 2.1. Study Area, Sampling and Data Collection

From 2009 to 2023, processed samples of whole blood from domestic dogs were submitted to INNO Veterinary Laboratories (Braga, Portugal), a veterinary diagnostic laboratory in North Portugal. The samples were collected from 286 veterinary medical centres (i.e., clinics and hospitals) from all of the regions of mainland Portugal. Along with the samples, a laboratory requisition was received, which included the complete clinical information for each dog, particularly their breeds, sexes, ages, vaccination and prophylactic statuses, clinical suspicion/clinical signs and requested analyses.

As inclusion criteria, only samples of dogs (*Canis lupus familiaris*) with clinical suspicion of CanL or those residing in areas where the disease is endemic, cohabiting with another potentially infected animals or having recently returned from a trip to an endemic region, were included. All biological samples were verified for analysis suitability (haemolysis, lipemia, correct anticoagulant ratio to sample volume, date of collection and other factors that may affect the result of the test).

### 2.2. Blood Analysis and Serological Tests

The tests on complete blood count (CBC), red blood cells (RBC), packed cell volume (PCV), platelets (PLT) and white blood cells (WBC) were conducted using the Sysmex XT-2000iV Analyzer (Sysmex Corporation, Kobe, Japan), with platelet count confirmation being carried out through blood smear stained with Giemsa. The measurements of total protein (TP), urea (UREA), creatinine (CREA) and alanine aminotransferase (ALAT) were performed using the Cobas^®^ 6000 Analyzer (c501) (Roche, Grenzach-Wyhlen, Germany). Protein electrophoretic separation was carried out using the method and materials recommended by the Sebia^®^ Minicap Flex Piercing Analyser (Sebia, Norcross, GA, USA) for capillary electrophoresis.

The samples were tested using the enzyme-linked immunosorbent assay (ELISA)-based quantitative assay, the LEISCAN^®^ *Leishmania* ELISA Test (Ecuphar, Barcelona, Spain), to detect specific antibodies to *Leishmania infantum* antigens in dogs. This test has a sensitivity and specificity of 95.3% and 99.8%, respectively [26,42,43,44]. The ELISA methodology is accredited and recommended by the World Organization for Animal Health (WOAH) as one of the reference methods for diagnosing and monitoring infection and disease [45]. The ranges adopted for classifying the animals were those defined by the test: negative (Rz ≤ 0.9), inconclusive (0.9 < Rz ≤ 1.1), low positive (1.1 < Rz ≤ 1.5), positive to high positive (1.5 < Rz ≤ 3) and very high positive (Rz > 3). The ratio (Rz) corresponds to the absorbance ratio of the positive control to the sample absorbance. These intervals were maintained in this manner (instead of being classified merely as positive, for example) for a subsequent analysis of the correlation of *Leishmania* results with other parameters to examine whether the ELISA test ratio (Rz) had an association with any of these.

### 2.3. Statistical Analysis

All of the data were available in digital format in Clinidata^®^ (Clinidata XXI version 5.3.25, Maxdata Software, S.A., Carregado, Portugal) and transferred to Microsoft Excel^®^ (Microsoft, Redmond, WA, USA) sheets. Statistical analysis was conducted using the JMP^®^, version 14.3 SAS Institute, Cary, NC, USA, 1989–2023 SAS and MedCalc^®^ Statistical Software version 20.006 (MedCalc Software Ltd., Ostend, Belgium, 2021). To study the differences between the observed and expected frequencies of categories of a field, nonparametric tests were used, including the binomial test or the Chi-Square test for one-sample analysis, depending on the number of categories in the categorical field. For comparisons among three or more independent groups, the Kruskal–Wallis test was utilised, followed by the Dunn–Bonferroni post hoc test for multiple comparisons when appropriate. Additionally, logistic regression with Tikhonov regularisation was employed to identify breeds with a higher predisposition for testing positive while accounting for a disproportional representation of breeds in the study [46,47,48]. The sample parameters were categorised into two great groups:

Qualitative variables: These involve the district, region (NUTS2), sex, breed and age categories (puppy: <1 year old; young: 1 to <2 years old; adult: 2 to <6 years old; senior: 6 to <11 years old; old: ≥11 years old).

Quantitative variables: These involve CBC parameters such as RBC, PCV, PLT, leukogram (WBC), neutrophils (NEU), lymphocytes (LYM), monocytes (MON), eosinophils (EOS), basophils (BASO), biochemical data (CREA, UREA, ALAT and TP), protein profile (albumin (ALB), alpha 1 and 2, beta 1 and 2, and gamma globulins), albumin/globulin ratio (ALB/GLOB) and results from the quantitative ELISA test classified according to test absorbance (negative, inconclusive, low positive, positive to high positive and very high positive).

## 3. Results

### 3.1. Qualitative Variables

#### 3.1.1. Descriptive Data

Of the total of 2910 animals included in this study, only 2801 animals were analysed after the removal of inconclusive ELISA results (*n* = 109, 3.8%). From the 2801 animals included, 38.8% tested negative (*n* = 1129), and 57.5% tested positive (*n* = 1672).

#### 3.1.2. Geographical Distribution

**Figure 1:**
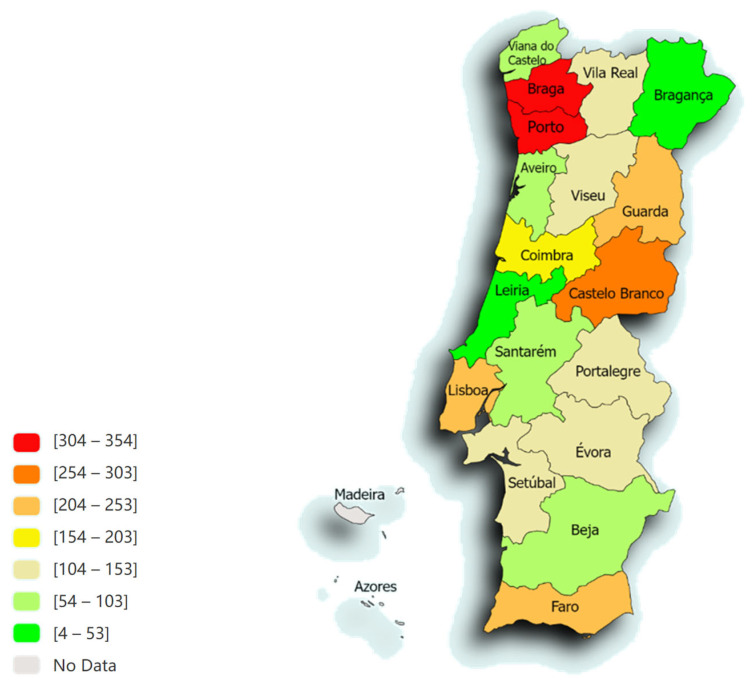
The spatial distribution, according to the distribution of animals from the different districts of Portugal, of the 2801 animals admitted to the LEISCAN^®^ *Leishmania* ELISA Test (map drawn in paintmaps.com; accessed on 10 June 2024).

Figure 1 represents the distribution of animals in the different districts of Portugal. The majority of samples originated from Porto (12.6%; *n* = 354), Braga (11.8%; *n* = 330), Castelo Branco (9.8%; *n* = 273) and Lisboa (8.9%; *n* = 249).

The distribution was divided using the NUTS2 (Nomenclature of Territorial Units for Statistics) regions of mainland Portugal as follows: 33.2% in the north region (*n* = 930), 32.4% in the centre (*n* = 906), 10.4% in Alentejo (*n* = 290), 8.1% in Algarve (*n* = 226), 7.9% in Greater Lisbon (GL) (*n* = 220), 4.2% in Oeste e Vale do Tejo (OVT) (*n* = 118) and 4.0% in Península de Setúbal (PdS) (*n* = 111). The region with the highest prevalence of positive cases was the OVT region (68.6%), followed by the Alentejo region (68.3%).

The Chi-Square test revealed a statistically significant relationship between the region and the negative and positive outcomes of the LEISCAN^®^ *Leishmania* ELISA Test (*p* < 0.001). The average prevalence of the disease in this study across different regions was 59.7%, with an average percentage variation of 9.0% (ranging from 42.3% to 68.6%). The Chi-Square test also indicated a statistically significant relationship between region and prevalence/positivity (*p* < 0.001). Table 1 illustrates the percentage of positives in the current study during the years 2009 to 2023 across the geographical regions of mainland Portugal.

Table 2 illustrates the percentage of positives in the current study during the years 2014 to 2023 across the regions of mainland Portugal.

**Figure 2:**
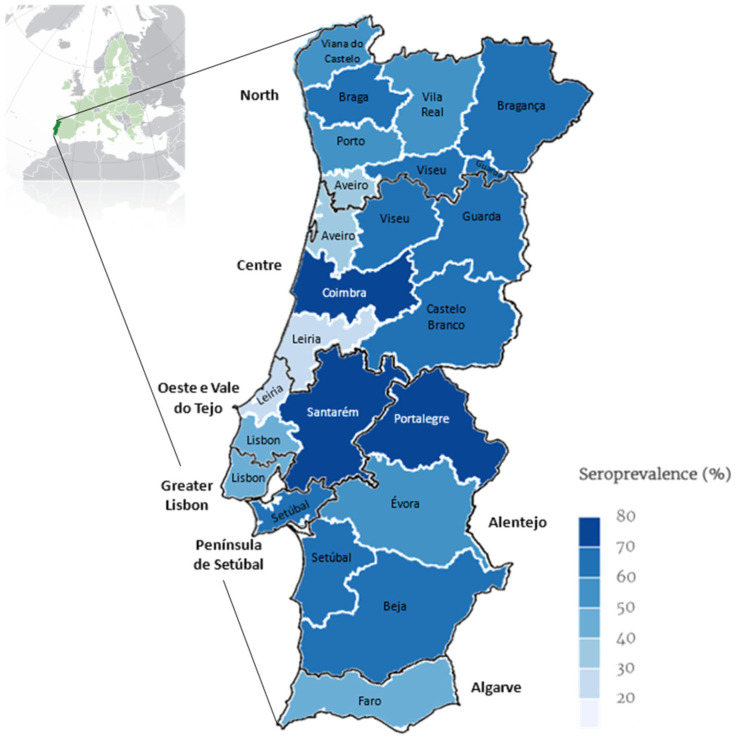
Map of continental Portugal showing categorical representation of average percentage of prevalence rate over 15 years (2009–2023) for *Leishmania* seroprevalence determined by ELISA per district and NUTS2 (map drawn in mapinseconds.com; accessed on 10 June 2024).

Figure 2 displays the average percentage of positivity over a 15-year period in mainland Portugal (2009–2023).

#### 3.1.3. Sex

From the 2801 animals analysed, 964 (34.4%) were females and 1837 (65.6%) were males. Table 3 represents the percentages of positive and negative tests according to sex. The differences observed between animal sexes were not significant (*p* = 0.082).

#### 3.1.4. Breed

Regarding breed, our study population comprised animals from 83 different dog breeds that included the following: 1365 mixed-breed dogs (48.7%), 306 Labrador Retrievers (10.9%), 147 German Shepherds (5.3%), 85 Boxers (3.0%), 72 Portuguese Podengos (2.6%), 48 Miniature Pinschers (1.7%), 45 Epagneul Bretons (1.6%), 43 Beagles (1.5%), 38 Cocker Spaniels (1.4%), 37 French Bulldogs (1.3%), 37 American Pit Bull Terriers (1.3%), 33 Poodles (1.2%), 32 Estrela Mountain Dogs (1.1%), 31 Golden Retrievers (1.1%), 26 Siberian Huskies (0.9%) and 68 other breeds.

For statistical analysis, the mixed-breed category was excluded. Despite the significant difference among breeds in the LEISCAN^®^ *Leishmania* ELISA indicated by the Kruskal–Wallis test (*p* < 0.001), no significant associations were found by the Dunn–Bonferroni test; all adjusted *p*-values were above 0.05. Therefore, there is insufficient evidence of a significant relationship between dog breed and the negative and positive outcomes of the LEISCAN^®^ *Leishmania* ELISA Test at the 0.05 significance level. However, logistic regression with Tikhonov regularisation suggested Pugs (coef.: 1.22), Rottweilers (coef.: 1.11), English Bulldogs (coef.: 0.97), Basset Hounds (coef.: 0.97) and Jack Russel Terriers (coef.: 0.96) as having a higher predisposition for testing positive. Among the breeds with the highest prevalence of positives, Labrador Retrievers had 11.2% (*n* = 187), German Shepherds had 5.0% (*n* = 83), Boxers had 3.8% (*n* = 64) and Portuguese Podengos had 2.8% (*n* = 47).

#### 3.1.5. Age

From the 2801 animals that were analysed, age data were available for only 2513 animals, as for 288 animals (10.3%), requisition documents did not specify their age and were thus excluded from certain analytical processes. The age distribution among these 2513 animals ranged from ≤1 year (6 months) to 23 years, with an average age of 7.2 ± 3.9 years. A total of 1.2% (*n* = 33) were puppies, 2.6% (*n* = 73) were young, 30.4% (*n* = 850) were adults, 41.0% (*n* = 1149) were seniors and 14.6% (*n* = 408) were old.

Table 4 displays the percentages of positive and negative results according to age group. The result of the Spearman correlation analysis indicated a significant correlation between age and the negative and positive outcomes of the quantitative ELISA test (*p* < 0.001). A significance level of *p* = 0.004 was also observed in the Cochran–Armitage test for the frequency of seropositivity according to the life stage of the individual studied, indicating that the disease predisposition is associated with age. This result suggests that age influences the likelihood of positive results in the serological test, with adult and senior age groups showing a higher incidence of seropositivity than others.

### 3.2. Quantitative Variables

#### 3.2.1. Blood and Biochemical Data

Table 5 presents the values of blood and biochemical data recovery from 2801 dogs included in this study, both negative and positive.

**Figure 3:**
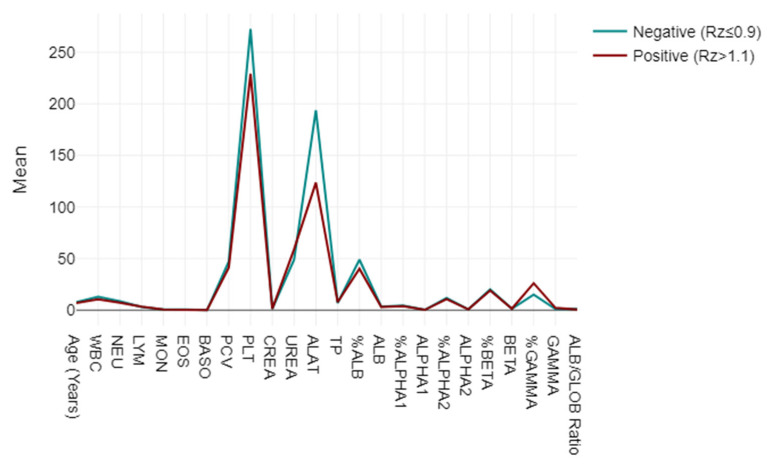
Mixed-model ANOVA findings: blood and biochemical markers, categorised based on their responses to LEISCAN^®^ *Leishmania* ELISA Test as either negative (Rz ≤ 0.9) or positive (Rz > 1.1).

A mixed-model ANOVA was utilised to evaluate blood and biochemical markers, categorising them based on their responses to the LEISCAN^®^ *Leishmania* ELISA Test as either negative (Rz ≤ 0.9) or positive (Rz > 1.1). The analysis highlighted strong associations in the key parameters, including lower WBC, NEU, PCV and PLT levels in positive cases, alongside elevated UREA and GAMMA globulin levels, suggesting impactful physiopathological correlations. The ALB/GLOB ratio also varied, indicating potential diagnostic utility (*p* < 0.001) (Figure 3).

#### 3.2.2. Receiver Operating Characteristic (ROC) Curve Analyses

**Figure 4:**
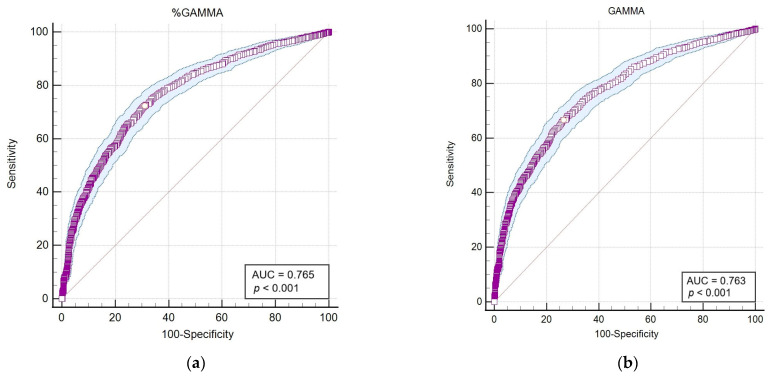

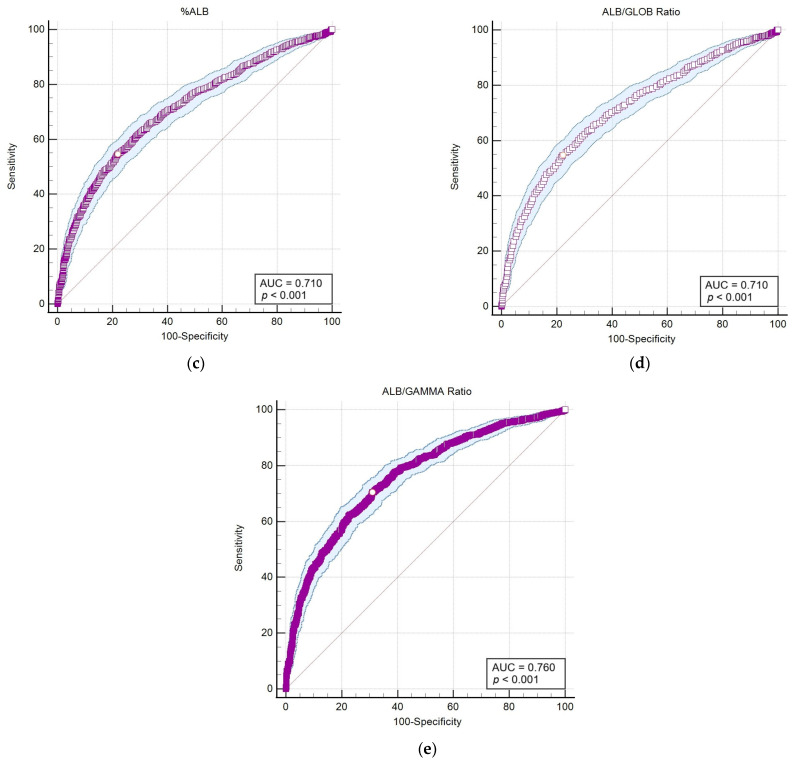
The area under the curve–receiver operating characteristic (AUC-ROC) of parameters that showed the highest correlation and statistical significance (*p* < 0.001) with the LEISCAN^®^ *Leishmania* ELISA Test. (**a**) The AUC-ROC of %GAMMA; (**b**) the AUC-ROC of GAMMA (g/dL); (**c**) the AUC-ROC of %ALB; (**d**) the AUC-ROC of the ALB/GLOB ratio (g/dL); (**e**) the AUC-ROC of the ALB/GAMMA ratio (g/dL).

Following the application of Spearman’s statistical correlation analysis, haematological and biochemical markers exhibiting the highest correlations and statistical significance (*p* < 0.001) with outcomes from the LEISCAN^®^ *Leishmania* ELISA Test were identified for detailed analysis. Subsequently, an area under the curve–receiver operating characteristic (AUC-ROC) analysis was performed to evaluate the diagnostic sensitivity and specificity of these selected parameters for the positive serology and consequent infection of *Leishmania* (Table 6 and Figure 4).

The AUC-ROC analysis serves as an essential statistical instrument for assessing diagnostic test performance. It notably emphasises the GAMMA (%) and ALB/GAMMA ratio as particularly valuable metrics, offering high sensitivity and specificity for the diagnosis of leishmaniosis.

## 4. Discussion

Although *Leishmania* spp. can infect a variety of vertebrate species, dogs are the main reservoir for zoonotic *L. infantum* in the Mediterranean region [6,24]. In Portugal, canine epidemiological studies in different regions have been performed over the years, with the last national seroepidemiological survey being conducted in 2021 [6]. However, no studies have explored the relationships between epidemiological and clinicopathological parameters, nor have any thoroughly described clinicopathologic abnormalities and their clinical usefulness in Portugal, a country where the disease is endemic. In the present study, the authors present an update on CanL in Portugal and its relationship with its clinicopathological parameters. To the authors’ knowledge, this is the first extensive work performed in Portugal and Europe.

The analysis of the data reveals significant physiological differences between groups based on various blood and biochemical markers, highlighting the influence of age and specific conditions on these parameters. With statistically significant *p*-values indicating robust differences across markers such as WBC, NEU, LYM and the ALB/GLOB ratio, our findings underscore the potential of these parameters as diagnostic tools or indicators of physiological responses. This study contributes to the understanding of the complex interactions between physiological markers and specific health conditions, offering insights for future research and clinical practise. The Spearman correlation analysis reveals a range of relationships between age, various blood parameters (like WBC, NEU, LYM, etc.) and the LEISCAN^®^ *Leishmania* ELISA Test. While most correlations with age and blood parameters are weak (close to 0), indicating little to no linear relationship, some notable exceptions exist, such as a modest positive correlation between age and certain parameters like PLT and the ALB/GLOB ratio. The negative correlations, though generally weak, suggest slight inverse relationships, such as age with BASO and PCV. The LEISCAN^®^ *Leishmania* ELISA Test shows a pattern of weak negative correlations with most blood parameters, suggesting that as the condition progresses from negative to positive, some blood parameters may decrease slightly. Overall, the correlations provide insights but also highlight the complexity of relationships between age, blood parameters and disease status, warranting further investigation for a clearer understanding. Consequently, the AUC-ROC analysis emerges as a crucial statistical tool for evaluating the efficacy of diagnostic tests. It particularly underscores the significance of the GAMMA (%) and ALB/GAMMA ratio, identifying them as highly valuable metrics that deliver high sensitivity and specificity in diagnosing leishmaniosis.

### 4.1. Descriptive Data and Geographical Distribution

In the present study, 59.7% of the analysed animals were positive for antibodies to *Leishmania* spp., indicating exposure to the parasite. However, this rate does not reflect the true seroprevalence in the general canine population, as the dogs included had clinical suspicions of the disease. Additionally, serological tests can detect antibodies in animals that have been exposed to the parasite but may not necessarily have an active infection. This contrasts with a previous national study that revealed an overall true seroprevalence of 12.5% in dogs [6,49] and 4.8% in humans [50]. The data obtained in this study challenge the previously held belief that the *Leishmania* frequency in Portugal was predominantly confined to the southern regions and in small endemic *foci* in the north of the country [16,51,52,53], unveiling a broader geographical distribution across the mainland, which aligns with findings from the most recent epidemiological studies [6,7]. The Oeste e Vale do Tejo and Alentejo regions reported the highest positivity rates, with 68.6% and 68.3%, respectively, highlighting the north region, which surprisingly exhibits a notable prevalence of 60.1%, indicating that no geographical area is free from CanL [52,54]. These findings support seroprevalence studies in humans, where sub-regions in the northern part of the country exhibited the highest prevalences, which may be the result of geographical shifts in parasite circulation due to climate change [39,55].

There is a significant correlation between the geographical region and *Leishmania* infection outcomes (*p* < 0.001), highlighting the influence of location on disease prevalence. This finding is pivotal as it aligns with the Stockholm Paradigm, which posits that pathogens can expand beyond their traditional ecological niches, adapting to new hosts and environments [38,39,56].

### 4.2. Sex

The distribution of positive samples between females (34.4%) and males (65.6%) in this study presents a substantial skew towards males. However, that difference was not statistically significant (*p* = 0.082). This finding suggests that, contrary to certain diseases where sex might play a role in susceptibility due to hormonal, genetic or behavioural factors, the *Leishmania* infection values in this population do not significantly differ between males and females [57,58].

The lack of significant difference in infection rates by sex supports the notion that preventive measures and clinical interventions should be uniformly applied across sexes, focusing instead on other risk factors. The observed data are consistent with what has been reported over the past two decades [57].

### 4.3. Breed

The significant diversity observed among breeds, with mixed breeds constituting a significant portion (48.7%), underlines the heterogeneity of the study population. Despite this diversity, the Kruskal–Wallis test indicated a significant difference among breeds in their response to the LEISCAN^®^ *Leishmania* ELISA Test (*p* < 0.001). However, the Dunn–Bonferroni post hoc test showed no significant associations between particular breeds and the ELISA outcomes, indicating that despite there being breed diversity, it does not significantly influence the likelihood of a positive or negative test result. Our results confirm that the seroprevalence of *L. infantum* among different canine breeds is not associated with antibody titres, which aligns with the findings of authors from recent studies [57,59,60].

### 4.4. Age

The age distribution was bimodal, with the highest prevalence of the disease occurring at 2–5 years of age and a secondary peak occurring at 6 years or over. The age analysis revealed a significant correlation between age and *Leishmania* infection outcomes (*p* < 0.001), with a notable predisposition towards adult and senior dogs. This trend is further supported by the Cochran–Armitage test, highlighting a significant association between age and seropositivity frequency (*p* < 0.004), suggesting that older animals are more likely to develop the disease. The clear correlation between age and infection frequency emphasises the need for targeted surveillance and preventive measures for older dogs, who are at a higher risk. However, it is crucial to implement preventive measures from an early age. The observed data are consistent with what has been reported [6,57,61].

### 4.5. Quantitative Variables

Spearman’s correlation analysis (Supplementary Materials: Table S1) was performed on the data obtained. To facilitate interpretation, the authors chose to expound the most salient associations, with correlations manifesting robust (either positive or negative) relational magnitudes underscored by their statistical significance.

Drawing from the Spearman correlation coefficients at hand, the interpretations for the most pronounced positive correlations are as follows:

- PCV vs. ALB (%) (r = 0.59; *p* < 0.001) and PCV vs. ALB (r = 0.61; *p* < 0.001): Both show a moderate positive correlation, suggesting that as the packed cell volume increases, there is an increase in both the percentage and absolute levels of albumin.
- TP vs. GAMMA (%) (r = 0.59; *p* < 0.001) and TP vs. GAMMA (g/dL) (r = 0.71; *p* < 0.001): Both show a moderate to strong positive correlation, suggesting that as the total protein level increases, there is an increase in both the percentage and absolute levels of gamma globulins.
- CREA vs. UREA (r = 0.53; *p* < 0.001): There is a moderate positive correlation, suggesting that as the creatinine increases, there is an increase in urea levels.

Such positive correlations may be indicative of typical physiological interactions; alternatively, they might signal specific pathological states when deviating from established normative ranges.

The following is an interpretation of the most significant negative correlations, as determined by the provided correlation coefficients (r):

- ALB/GLOB Ratio vs. GAMMA (%) (r = −0.77; *p* < 0.001) and ALB/GLOB Ratio vs. GAMMA (g/dL) (r = −0.77; *p* < 0.001): Both show a similarly strong inverse relationship, indicating that higher albumin/globulin ratios are associated with lower overall gamma globulin levels.
- GAMMA (%) vs. ALB (%) (r = −0.77; *p* < 0.001) and GAMMA (g/dL) vs. ALB (%) (r = −0.77; *p* < 0.001): There is a strong negative correlation, indicating that a higher percentage of albumin in the blood is associated with lower overall gamma globulins.
- ALB vs. GAMMA (%) (r = −0.54; *p* < 0.001) and ALB vs. GAMMA (g/dL) (r = −0.46; *p* < 0.001): These indicate a moderate inverse relationship, suggesting that higher total albumin levels are associated with lower overall gamma globulins.
- PCV vs. GAMMA (%) (r = −0.50; *p* < 0.001) and PCV vs. GAMMA (g/dL) (r = −0.45; *p* < 0.001): There is a moderate negative correlation, suggesting that higher packed cell volume (PCV) values are associated with lower overall gamma globulins.
- MON vs. CREA (r = −0.22; *p* < 0.001): There is a weak negative correlation, indicating that as the monocyte counts increase, the creatinine levels tend to decrease slightly.
- PLT vs. GAMMA (g/dL) (r = −0.23; *p* < 0.001) and PLT vs. GAMMA (%) (r = −0.22; *p* < 0.001): Both indicate weak negative correlations, suggesting that higher platelet counts are associated with slightly lower gamma globulin levels and percentages.

Positive correlations may be indicative of typical physiological interactions; alternatively, they might signal specific pathological states when deviating from established normative ranges. These correlations highlight important relationships between different blood markers and liver functions, reflecting how biological variables interact. Strong and significant correlations (*p* < 0.001) are particularly relevant for understanding the pathophysiology underlying these measurements.

#### 4.5.1. Serological Correlations

In the context of the LEISCAN^®^ *Leishmania* ELISA Test, we conducted an analysis to explore serological correlations based on age, blood and biochemical data from 2801 animals. Table 7 serves as a critical reference for understanding the complex interactions between various physiological markers used in our study and *Leishmania* infection.

The data reveal significant correlations between various haematological and biochemical parameters and *Leishmania* seropositivity. Gamma globulins (GAMMA) and total protein (TP) show strong and moderate positive correlations, respectively, while urea (UREA) and creatinine (CREA) exhibit weaker positive correlations. Conversely, monocytes (MON), basophils (BASO), alanine aminotransferase (ALAT), eosinophils (EOS), neutrophils (NEU), total leukocytes (WBC), platelets (PLT), albumin (ALB), and packed cell volume (PCV) demonstrate significant negative correlations with seropositivity. The overall trend indicates that *Leishmania*-seropositive dogs tend to have higher levels of gamma globulins and total protein but lower levels of several other haematological and biochemical parameters. This suggests a complex interaction between age, immune response and disease susceptibility.

#### 4.5.2. Haematological and Serum Protein Markers

Regarding leukocyte profiling, a similar inverse relationship is noted in the WBC count, with a correlation coefficient of −0.17, indicating a weak yet statistically significant negative association with positive ELISA outcomes. This trend is paralleled in the NEU counts, which mirror the WBC results in both magnitude and significance. The MON and BASO counts display minimal inverse correlations with the ELISA results with statistical significance. The EOS counts are weakly negatively correlated, suggesting a trend towards negative ELISA outcomes with increased eosinophil counts. A more pronounced negative correlation is observed with PCV, where Spearman’s rho of −0.29 suggests that as the PCV levels decrease (or are lower), the likelihood of a positive test for *Leishmania* increases. The PLT counts also demonstrate a negative correlation with the ELISA outcomes, supporting the hypothesis that specific haematological parameters can play a crucial role in the understanding of *Leishmania* infection. We hypothesise that in the early stages of leishmaniosis, dogs exhibit lower leukocyte counts, including polymorphonuclear cells (PMNC) and monocytes, with mild leukocytosis with neutrophilia or monocytosis developing as the disease progresses. Additionally, eosinophilia, occasionally reported in leishmaniosis and associated with parasitic infestation or an allergic component, could explain the weak negative correlation between eosinophil counts and ELISA outcomes. The pronounced negative correlation between PCV and ELISA outcomes may indicate that as the PCV levels decrease, reflecting moderate non-regenerative anaemia often seen in advanced stages of leishmaniosis, the likelihood of a positive ELISA test increases. This aligns with our hypothesis that dogs with lower PCV levels are more likely to test positive for *Leishmania* due to the disease’s impact on blood cell production. These observations align with what has been previously observed in other studies [62,63,64].

In the data, it is possible to observe a nuanced interplay between the serum protein levels and ELISA outcomes, employing the LEISCAN^®^ ELISA methodology for serodiagnosis. These significant correlations between the serum protein levels and ELISA outcomes reveal a complex biochemical interplay. Elevated TP levels are moderately positively correlated with ELISA positivity, indicative of an active humoral immune response to *Leishmania*, suggesting a substantial immunologic interaction with the pathogen, with elevated serum proteins being part of the inflammatory response. In contrast, both the percentage of ALB and the ALB/GLOB ratio exhibit moderate negative correlations, pointing to an inverse relationship between specific protein distribution patterns and serological evidence of infection, where albumin decreases and globulins increase due to the inflammatory response and antibody production. These findings are consistent with the available literature indicating the typical serum protein profiles in infection [32,65]. This suggests that certain biochemical profiles, particularly those involving ALB and GLOB ratios, may be inversely associated with the likelihood of infection.

Moreover, we observed a moderate positive correlation in the percentages of GAMMA and absolute GAMMA with ELISA positivity, underscoring the critical role of specific immunoglobulins in the disease’s pathophysiology and the adaptive immune system’s response. Additionally, our analysis extended to other serum proteins such as ALPHA 1 (%), ALPHA 1 (g/dL), ALPHA 2 (%), ALPHA 2 (g/dL), BETA (%) and BETA (g/dL), which showed correlations ranging from very weak to weak with ELISA outcomes, each with distinct statistical significance. This suggests a nuanced and complex relationship between serum protein profiles and *Leishmania* seropositivity, illustrating the diverse ways in which protein fractions may be linked to the presence of *Leishmania* antigens [29,66].

#### 4.5.3. Renal and Hepatic Biochemical Markers

In our study, we observed no statistical correlation of azotaemia across various LEISCAN^®^ *Leishmania* ELISA serological groups ranging from negative (Rz ≤ 0.9) to very high positive (Rz > 3). Interestingly, the creatinine levels showed a very weak positive correlation with ELISA positivity, suggesting that renal function, as indicated by creatinine levels, may only marginally impact the serological detection of *Leishmania*. Furthermore, the urea levels were weakly positively correlated, while ALAT, a marker of hepatic damage, exhibited a weak negative correlation with the ELISA outcomes.

These findings suggest a relatively low prevalence of cases in the advanced stages of the disease, characterised by elevated ALAT, UREA and/or CREA levels, primarily due to renal dysfunction or hepatic failure. This observation is contrary to the expectation set by many published studies [67,68,69,70,71], which often associate kidney disease with glomerular damage due to the deposition of immune complexes, leading to a progressive reduction in the perfusion of the peritubular capillaries and subsequent tubular and interstitial damage [53]. The lack of a strong association between high concentrations of circulating blood antibodies to *Leishmania* and increased levels of creatinine and/or urea in our study could be attributed to the large sample size or the relatively low sensitivity of serum creatinine and urea as early indicators of kidney disease [65,71,72]. These analytes mainly reflect filtration capacity rather than specific injury markers, which may explain why this serious disorder is infrequently described in the literature, likely because the disease is typically detected in less advanced phases before significant lesions develop [65,71,73,74,75,76].

In light of these observations, urinary biomarkers of proteinuria, such as Cystatin C (CisC) and neutrophil gelatinase-associated lipocalin (NGAL), emerge as more sensitive and specific indicators of glomerular and tubular damage, representing the optimal choice for renal evaluation [74,77]. A possible use in routine clinical practise, which is widely available, is symmetric dimethylarginine (SDMA), which may be a useful adjunct to serum creatinine (sCr) and the urine protein/creatinine ratio (Up/c), aligning with the International Renal Interest Society (IRIS) guidelines, established by the LeishVet group, for the early detection of CanL-associated nephropathy [8,71,77,78,79].

#### 4.5.4. Planetary Health in Leishmaniosis

Implementing a “One Health” approach in the fight against leishmaniosis includes integrated surveillance of human and animal health, vaccination campaigns, vector control, and community education on prevention and treatment. This strategy enhances the effectiveness of interventions and promotes sustainability in public and environmental health, which is crucial for managing zoonotic diseases in a connected world. The importance of this approach is reinforced by the Planetary Health framework, which acknowledges the interconnectedness of human health and natural systems, and the Stockholm Paradigm, which emphasises the ecological and evolutionary interactions in disease dynamics. Integrating these principles is vital for understanding and addressing the complex epidemiology of leishmaniosis, involving various hosts, vectors, and environmental factors, ultimately fostering a sustainable and health-conscious interaction among humans, animals, and the environment [80,81].

#### 4.5.5. Recommendations

For future articles investigating the epidemiology and clinicopathology of vector-borne diseases, such as canine leishmaniosis, it is imperative to adopt a holistic and integrative research methodology. This includes advanced statistical techniques and comprehensive geographic information systems (GIS) to analyse environmental and climatic impacts on disease vectors. Future studies should consider incorporating molecular diagnostics with conventional serological assays to improve detection accuracy. Collaboration with global health institutions and adherence to the One Health approach will be essential in understanding zoonotic diseases within the broader context of interconnected human, animal and environmental health. Detailed analyses of regional variations and specific risk factors are vital for developing targeted control measures and informing policy decisions. In this study, the authors regret not obtaining data on the urine protein/creatinine ratio (Up/c) and symmetric dimethylarginine (SDMA), which could have provided crucial insights into the understanding of the disease’s impact on renal function. Future research should incorporate these parameters to enhance the interpretation and management of leishmaniosis.

## 5. Conclusions

In this study, the authors analyse the seroprevalence and geographical distribution of *Leishmania* infection across mainland Portugal, challenging previous perceptions that the disease is confined to specific regions. The analysis, based on a dataset of 2801 dogs, shows a 59.7% positivity rate for *Leishmania* antibodies, indicating a widespread distribution of the disease, potentially influenced by shifts in parasite circulation due to climate change. However, these findings do not represent the true seroprevalence in the general canine population, as the included dogs may be biassed because the submitted samples come from animals with a higher suspicion of *Leishmaniosis* than the general population. As a result, these outcomes significantly differ from earlier studies in Portugal, which used random sampling techniques and reported an overall true seroprevalence of 12.5% in dogs and 4.8% in humans. This highlights the critical need for enhanced surveillance and preventive measures across all regions of Portugal.

Our analysis revealed significant variances in seroprevalence, notably in the centre and northern regions, challenging prior studies and suggesting a more extensive disease spread. These data are further corroborated by a significant correlation between geographical location and infection outcomes, aligning with the Stockholm Paradigm that pathogens are capable of expanding beyond their traditional ecological confines. Dog age emerged as a significant factor in *Leishmania* infection, with a noted bimodal predisposition towards adult and senior dogs.

Despite the lack of a statistical correlation between the azotaemia and ELISA serological groups, the observed weak positive correlations with the creatinine and urea levels suggest renal function’s limited impact on *Leishmania* detection. This challenges the traditional linkage between kidney disease and *Leishmania* infection, which has traditionally been attributed to glomerular damage due to immune complex deposition, highlighting the importance of considering renal and hepatic function markers as indirect indicators of disease severity. The data obtained in this study emphasise the necessity of considering renal and hepatic markers in the broader context of disease stage distribution within the population.

Significantly, this study underscores the diagnostic value of examining albumin and globulin ratios, with the albumin percentage and the albumin/globulin ratio (ALB/GLOB) showing moderate negative correlations with serological evidence of infection. The application of AUC-ROC analysis has further identified the albumin/gamma globulin ratio (ALB/GAMMA) as a valuable diagnostic metric, offering high sensitivity and specificity for *Leishmania* detection and the diagnosis of CanL.

In conclusion, this study elucidates the complex seroprevalence patterns and biochemical markers associated with *Leishmania* infection, underscoring the imperative for a sophisticated approach to diagnostics, surveillance and disease management. Our findings significantly enrich the current understanding of *Leishmania*, advocating for an approach that integrates localised research efforts within a One Health framework to efficiently address the disease’s multifaceted impact.

## Figures and Tables

**Table 1:** Negative and positive LEISCAN^®^ *Leishmania* ELISA Test by regions in the 2801 animals included in this study.

		Negative and Positive LEISCAN^®^ *Leishmania* ELISA Test				
		Negative (Rz ≤ 0.9)		Positive (Rz > 1.1)		Total
		*n*	% within Region	*n*	% within Region (Prevalence)	*n*
**NUTS2 Regions**	North	371 (13.3%)	39.9%	559 (20.0%)	60.1%	930 (33.2%)
	Centre	334 (11.9%)	36.9%	572 (20.4%)	63.1%	906 (32.4%)
	OVT	37 (1.3%)	31.4%	81 (2.9%)	68.6%	118 (4.2%)
	Greater Lisbon	127 (4.5%)	57.7%	93 (3.3%)	42.3%	220 (7.9%)
	PdS	40 (1.4%)	36.0%	71 (2.5%)	64.0%	111 (4.0%)
	Alentejo	92 (3.3%)	31.7%	198 (7.1%)	68.3%	290 (10.4%)
	Algarve	128 (4.6%)	56.6%	98 (3.5%)	43.4%	226 (8.1%)
	Total	1129	40.3%	1672	59.7%	2801

OVT, Oeste e Vale do Tejo; PdS, Península de Setúbal.

**Table 2:** Evolution of positives over 10 years in mainland Portugal (2014–2023).

Evolution of Percentage of Positives over 10 Years in Mainland Portugal											
Regions	2014	2015	2016	2017	2018	2019	2020	2021	2022	2023	Mean
North	71.4%	61.4%	68.0%	57.8%	64.6%	68.0%	62.8%	56.4%	52.6%	54.0%	61.7%
Centre	36.6%	53.9%	66.7%	62.2%	68.1%	74.8%	66.4%	59.6%	64.8%	67.7%	62.1%
OVT	0.0%	80.0%	72.7%	85.7%	62.5%	77.8%	66.7%	61.1%	56.0%	79.2%	64.2%
Greater Lisbon	66.7%	50.0%	100%	44.4%	45.7%	45.2%	40.5%	30.4%	44.8%	35.1%	50.3%
PdS	55.6%	60.0%	0.0%	100%	50.0%	42.9%	46.2%	72.2%	76.9%	92.9%	59.7%
Alentejo	33.3%	92.3%	71.4%	70.2%	76.2%	58.6%	54.8%	72.0%	74.3%	65.9%	66.9%
Algarve	85.7%	42.9%	40.6%	20.0%	38.2%	62.5%	41.7%	42.9%	37.5%	44.4%	45.6%
Mean	49.9%	62.9%	59.9%	62.9%	57.9%	61.4%	54.1%	56.4%	58.1%	62.7%	-

OVT, Oeste e Vale do Tejo; PdS, Península de Setúbal.

**Table 3:** Negative and positive LEISCAN^®^ *Leishmania* ELISA Test by sex in the 2801 animals included in this study.

	LEISCAN^®^ *Leishmania* ELISA Test		
Sex	Negative (Rz ≤ 0.9)	Positive (Rz > 1.1)	Total
Female	410 (14.6%)	554 (19.8%)	964 (34.4%)
Male	719 (25.7%)	1118 (39.9%)	1837 (65.6%)
Total	1129 (40.3%)	1672 (59.7%)	2801 (100%)

**Table 4:** Negative and positive LEISCAN^®^ *Leishmania* ELISA Test by age group in 2513 dogs included in this study.

Age Group							
		Puppy (<1 Year Old)	Young (1 to <2 Years Old)	Adult (2 to <6 Years Old)	Senior (6 to <11 Years Old)	Old (≥11 Years Old)	Total
Negative and Positive LEISCAN^®^ *Leishmania* ELISA Test	Negative (Rz ≤ 0.9)	24 (1.0%)	48 (1.9%)	287 (11.4%)	459 (18.3%)	210 (8.4%)	1028 (40.9%)
	Positive (Rz > 1.1)	9 (0.4%)	25 (1.0%)	563 (22.4%)	690 (27.5%)	198 (7.9%)	1485 (59.1%)
	Total	33 (1.3%)	73 (2.9%)	850 (33.8%)	1149 (45.7%)	352 (16.6%)	2513 (100%)

**Table 5:** Blood and biochemical data recovery from 2801 dogs, both negative and positive, in the LEISCAN^®^ *Leishmania* ELISA Test.

				Negative (Rz ≤ 0.9) (*n* = 1129, 40.3%)	Low Positive (1.1 < Rz ≤ 1.5) (*n* = 222, 7.9%)	Positive to High Positive (1.5 < Rz ≤ 3) (*n* = 784, 28.0%)	Very High Positive (Rz > 3) (*n* = 666, 23.8%)	All Positives (*n* = 1672, 59.7%)		
Parameter (Units)	Reference Range	Minimum	Maximum	Median (IQR)	Median (IQR)	Median (IQR)	Median (IQR)	Below the Physiological Range, *n*	Within the Physiological Range, *n*	Above the Physiological Range, *n*
WBC (10^3^/μL)	6.0–17.0	1.3	971.5	10.5	9.6	9.6	9.1	<6.0 (217)	6.0–17.0 (1339)	>17.0 (116)
NEU (10^3^/μL)	3.0–11.8	0.4	95.7	7.0	6.4	6.4	6.0	<3.0 (87)	3.0–11.8 (1436)	>11.8 (147)
LYM (10^3^/μL)	1.0–4.8	0.1	903.5	1.9	1.9	2.0	1.8	<1.0 (205)	1.0–4.8 (1421)	>4.8 (44)
MON (10^3^/μL)	0.2–2.0	0.0	12.4	0.4	0.4	0.4	0.4	<0.2 (283)	0.2–2.0 (1362)	>2.0 (25)
EOS (10^3^/μL)	0.1–1.3	0.0	22.6	0.5	0.4	0.4	0.4	<0.1 (201)	0.1–1.3 (1353)	>1.3 (116)
BASO (10^3^/μL)	0.0–0.5	0.0	0.5	0.0	0.0	0.0	0.0	-	0.0–0.5 (1670)	>0.5 (0)
PCV (%)	37.0–55.0	4.8	70.0	48.2	46.8	43.1	39.4	<37.0 (563)	37.0–55.0 (974)	>55.0 (135)
PLT (10^3^/μL)	200–500	5.0	928.0	251.0	228.5	216.0	208.0	<200 (677)	200–500 (944)	>500 (46)
CREA (mg/dL)	0.6–1.4	0.2	30.3	1.0	1.0	1.0	0.9	<0.6 (178)	0.6–1.4 (1158)	>1.4 (330)
UREA (mg/dL)	19.3–55.6	5.9	613.3	36.0	39.4	38.6	37.3	<19.3 (58)	19.3–55.6 (1191)	>55.6 (423)
ALAT (U/L)	17–95	0.0	4016.0	42.5	40.2	36.9	35.5	<17.0 (138)	17–95 (1358)	>95 (149)
TP (g/dL)	5.5–7.2	2.6	16.1	6.8	7.0	7.4	7.9	<5.5 (63)	5.5–7.2 (636)	>7.2 (973)
ALB (%)	-	0.2	78.6	50.6	48.2	40.7	36.8	-	-	-
ALB (g/dL)	2.44–4.96	0.6	7.5	3.4	3.4	3.1	3.0	<2.44 (402)	2.44–4.96 (1266)	>4.96 (3)
ALPHA 1 (%)	-	0.1	25.1	4.4	4.4	3.9	3.7	-	-	-
ALPHA 1 (g/dL)	0.17–0.45	0.0	2.8	0.3	0.3	0.3	0.3	<0.17 (314)	0.17–0.45 (1183)	>0.45 (174)
ALPHA 2 (%)	-	1.0	44.0	11.0	11.0	10.4	10.0	-	-	-
ALPHA 2 (g/dL)	0.38–1.02	0.1	2.7	0.7	0.8	0.8	0.8	<0.38 (124)	0.38–1.02 (1179)	>1.02 (368)
BETA (%)	-	2.2	59.9	19.0	19.1	18.9	17.5	-	-	-
BETA (g/dL)	0.53–2.4	0.2	6.3	1.3	1.3	1.4	1.3	<0.53 (36)	0.53–2.4 (1514)	>2.4 (121)
GAMMA (%)	-	0.6	76.2	12.4	14.8	20.9	28.7	-	-	-
GAMMA (g/dL)	0.26–1.17	0.1	12.3	0.8	1.0	1.5	2.2	<0.26 (7)	0.26–1.17 (552)	>1.17 (1112)
ALB/GLOB ratio	0.86–1.93	0.1	3.7	1.0	0.9	0.7	0.6	<0.86 (1091)	0.86–1.93 (567)	>1.93 (13)

ALB (%), percentage of albumin; ALPHA 1 (%), percentage of alpha 1 globulins; ALPHA 1 (g/dL), absolute levels of alpha 1 globulins; ALPHA 2 (%), percentage of alpha 2 globulins; ALPHA 2 (g/dL), absolute levels of alpha 2 globulins; BETA (%), percentage of beta globulins; BETA (g/dL), absolute levels of beta globulins; GAMMA (%), percentage of gamma globulins; GAMMA (g/dL), absolute levels of gamma globulins.

**Table 6:** Receiver operating characteristic (ROC) curve analyses of markers with the highest correlations and statistical significance (*p* < 0.001) with outcomes from the LEISCAN^®^ *Leishmania* ELISA Test.

Parameter	Threshold	Diagnostic Sensitivity (%)	Diagnostic Specificity (%)	AUC	*p*-Value
GAMMA (%)	>15.6%	72.2	69.1	0.765	<0.001
GAMMA (g/dL)	>1.17 g/dL	66.6	73.6	0.763	<0.001
ALB (%)	≤41.5%	54.6	78.1	0.710	<0.001
ALB/GLOB ratio	≤0.71	54.6	77.8	0.710	<0.001
ALB/GAMMA ratio	≤2.99	70.4	69.1	0.760	<0.001

ALB (%), percentage of albumin; AUC, area under the curve; GAMMA (%), percentage of gamma globulins; GAMMA (g/dL), absolute levels of gamma globulins.

**Table 7:** Age, blood and biochemical data correlations from the 2801 animals included in this study with the LEISCAN^®^ *Leishmania* ELISA Test in descending order of correlation strength.

Parameter	Spearman’s rho Correlation (r)	Statistical Significance (*p*-Value)
GAMMA (g/dL)	0.45	<0.001
GAMMA (%)	0.45	<0.001
TP	0.30	<0.001
UREA	0.11	<0.001
CREA	0.04	0.031
MON	−0.05	0.024
BASO	−0.05	0.014
Age	−0.09	<0.001
ALAT	−0.14	<0.001
EOS	−0.16	<0.001
NEU	−0.16	<0.001
WBC	−0.17	<0.001
PLT	−0.19	<0.001
ALB	−0.23	<0.001
PCV	−0.29	<0.001
ALB (%)	−0.36	<0.001
ALB/GLOB Ratio	−0.36	<0.001
Other Serum Proteins (ALPHA 1 (%), ALPHA 1 (g/dL), ALPHA 2 (%), ALPHA 2 (g/dL), BETA (%), BETA (g/dL))	Varied	Varied

ALB (%), percentage of albumin; ALPHA 1 (%), percentage of alpha 1 globulins; ALPHA 1 (g/dL), absolute levels of alpha 1 globulins; ALPHA 2 (%), percentage of alpha 2 globulins; ALPHA 2 (g/dL), absolute levels of alpha 2 globulins; BETA (%), percentage of beta globulins; BETA (g/dL), absolute levels of beta globulins; GAMMA (%), percentage of gamma globulins; GAMMA (g/dL), absolute levels of gamma globulins.

## Data Availability

The data presented in this study are available upon request from the corresponding authors.

## Supplementary Materials

The following supporting information can be downloaded at https://www.mdpi.com/article/10.3390/pathogens13080635/s1, Table S1. Spearman correlation analysis: age, blood and biochemical data from 2801 dogs across negative and positive LEISCAN^®^ *Leishmania* ELISA Test.
