# Supplementary material for: Distribution of and Relationships between Epidemiological and Clinicopathological Parameters in Canine Leishmaniosis: A Retrospective Study of 15 Years (2009–2023)
Source: Pathogens. 2024 Jul 29;13(8):635. doi: 10.3390/pathogens13080635 (PMC11357036; doi:10.3390/pathogens13080635)
Supplement: Supplementary file 1 [file pathogens-13-00635-s001.zip › pathogens-3094305-supplementary.pdf]

Table S1. Spearman correlation analysis: age, blood and biochemical data from 2801 dogs across negative and positive LEISCAN® *Leishmania* ELISA Test.

		Age (Years)	WBC	NEU	LYM	MON	EOS	BASO	PCV	PLT	CREA	UREA	ALAT	TP	%ALB	ALB	%ALPHA1	ALPHA1	%ALPHA2	ALPHA2	%BETA	BETA	%GAMMA	GAMMA	ALB/GLOB Ratio	ELISA
Age (Years)	r		−0.03	0.01	−0.11	0.04	−0.08	−0.13	−0.07	0.14	−0.05	0.02	0.14	−0.08	0.01	−0.03	0.04	0.01	0.17	0.12	0.07	0.02	−0.08	−0.09	0.01	−0.09
	p		.145	.454	<.001	.078	<.001	<.001	<.001	<.001	.02	.227	<.001	<.001	.458	.106	.061	.786	<.001	<.001	<.001	.216	<.001	<.001	.461	<.001
WBC	r	−0.03	1.00	0.93	0.43	0.63	0.35	0.24	−0.02	0.23	−0.08	−0.04	0.00	−0.03	−0.04	−0.07	0.01	−0.02	0.19	0.16	0.1	0.06	−0.06	−0.06	−0.04	−0.17
	p	.145		<.001	<.001	<.001	<.001	<.001	.332	<.001	<.001	.044	.967	.125	.032	<.001	.637	.392	<.001	<.001	<.001	.002	.001	.002	.031	<.001
NEU	r	0.01	0.93	1.00	0.16	0.57	0.2	0.16	−0.05	0.24	−0.06	−0.02	0.02	−0.02	−0.09	−0.12	0.01	−0.01	0.24	0.21	0.1	0.07	−0.04	−0.04	−0.09	−0.16
	p	.454	<.001		<.001	<.001	<.001	<.001	.008	<.001	.001	.421	.31	.365	<.001	<.001	.566	.515	<.001	<.001	<.001	.001	.029	.033	<.001	<.001
LYM	r	−0.11	0.43	0.16	1.00	0.19	0.36	0.31	0.16	0.07	−0.01	−0.05	−0.03	−0.03	0.17	0.17	0.02	0.01	−0.06	−0.07	0.00	0.00	−0.1	−0.08	0.17	−0.03
	p	<.001	<.001	<.001		<.001	<.001	<.001	<.001	<.001	.686	.008	.145	.173	<.001	<.001	.414	.552	.001	<.001	.814	.924	<.001	<.001	<.001	.118
MON	r	0.04	0.63	0.57	0.19	1.00	0.11	0.26	−0.27	0.15	−0.22	−0.05	−0.08	0.06	−0.2	−0.22	−0.05	−0.05	0.19	0.2	0.02	0.03	0.12	0.11	−0.2	−0.05
	p	.078	<.001	<.001	<.001		<.001	<.001	<.001	<.001	<.001	.017	<.001	.002	<.001	<.001	.017	.015	<.001	<.001	.404	.116	<.001	<.001	<.001	.024
EOS	r	−0.08	0.35	0.2	0.36	0.11	1.00	0.16	0.28	0.11	0.06	−0.09	−0.02	−0.09	0.2	0.18	0.00	−0.04	−0.1	−0.15	0.12	0.07	−0.15	−0.14	0.2	−0.16
	p	<.001	<.001	<.001	<.001	<.001		<.001	<.001	<.001	.004	<.001	.429	<.001	<.001	<.001	.956	.034	<.001	<.001	<.001	.001	<.001	<.001	<.001	<.001
BASO	r	−0.13	0.24	0.16	0.31	0.26	0.16	1.00	0.03	0.04	−0.18	−0.09	0.00	0.03	0.04	0.05	−0.06	−0.05	−0.07	−0.06	−0.05	−0.03	0.01	0.02	0.04	−0.05
	p	<.001	<.001	<.001	<.001	<.001	<.001		.135	.03	<.001	<.001	.849	.191	.064	.008	.004	.007	.001	.004	.014	.1	.551	.442	.065	.014
PCV	r	−0.07	−0.02	−0.05	0.16	−0.27	0.28	0.03	1.00	0.03	0.13	−0.17	0.22	−0.18	0.59	0.61	0.08	0.02	−0.04	−0.13	0.00	−0.07	−0.5	−0.45	0.59	−0.29
	p	<.001	.332	.008	<.001	<.001	<.001	.135		.113	<.001	<.001	<.001	<.001	<.001	<.001	<.001	.285	.043	<.001	.807	<.001	<.001	<.001	<.001	<.001
PLT	r	0.14	0.23	0.24	0.07	0.15	0.11	0.04	0.03	1.00	−0.07	0.02	0.1	−0.2	0.16	0.06	0.06	−0.02	0.15	0.05	0.05	−0.04	−0.22	−0.23	0.16	−0.19
	p	<.001	<.001	<.001	<.001	<.001	<.001	.03	.113		<.001	.331	<.001	<.001	<.001	.001	.003	.264	<.001	.015	.009	.038	<.001	<.001	<.001	<.001
CREA	r	−0.05	−0.08	−0.06	−0.01	−0.22	0.06	−0.18	0.13	−0.07	1.00	0.53	0.03	−0.04	0.02	0.03	0.03	0.02	0.00	−0.03	0.06	0.03	−0.04	−0.04	0.02	0.04
	p	.02	<.001	.001	.686	<.001	.004	<.001	<.001	<.001		<.001	.202	.039	.34	.194	.123	.276	.811	.106	.002	.16	.037	.032	.343	.031
UREA	r	0.02	−0.04	−0.02	−0.05	−0.05	−0.09	−0.09	−0.17	0.02	0.53	1.00	0.05	−0.1	−0.09	−0.15	0.08	0.05	0.07	0.01	0.02	−0.03	0.02	−0.01	−0.09	0.11
	p	.227	.044	.421	.008	.017	<.001	<.001	<.001	.331	<.001		.014	<.001	<.001	<.001	<.001	.021	.001	.597	.324	.152	.213	.801	<.001	<.001
ALAT	r	0.14	0.00	0.02	−0.03	−0.08	−0.02	0.00	0.22	0.1	0.03	0.05	1.00	−0.13	0.24	0.21	0.05	0.02	0.06	0.01	−0.02	−0.07	−0.25	−0.23	0.23	−0.14
	p	<.001	.967	.31	.145	<.001	.429	.849	<.001	<.001	.202	.014		<.001	<.001	<.001	.006	.373	.002	.74	.321	.001	<.001	<.001	<.001	<.001
TP	r	−0.08	−0.03	−0.02	−0.03	0.06	−0.09	0.03	−0.18	−0.2	−0.04	−0.1	−0.13	1.00	−0.56	−0.06	−0.32	0.04	−0.22	0.21	−0.01	0.43	0.59	0.71	−0.56	0.3
	p	<.001	.125	.365	.173	.002	<.001	.191	<.001	<.001	.039	<.001	<.001		<.001	.002	<.001	.048	<.001	<.001	.626	<.001	<.001	<.001	<.001	<.001
%ALB	r	0.01	−0.04	−0.09	0.17	−0.2	0.2	0.04	0.59	0.16	0.02	−0.09	0.24	−0.56	1.00	0.82	0.11	−0.09	−0.04	−0.29	−0.24	−0.45	−0.77	−0.77	1.00	−0.36
	p	.458	.032	<.001	<.001	<.001	<.001	.064	<.001	<.001	.34	<.001	<.001	<.001		<.001	<.001	<.001	.037	<.001	<.001	<.001	<.001	<.001	<.001	<.001
ALB	r	−0.03	−0.07	−0.12	0.17	−0.22	0.18	0.05	0.61	0.06	0.03	−0.15	0.21	−0.06	0.82	1.00	−0.05	−0.08	−0.17	−0.2	−0.26	−0.26	−0.54	−0.46	0.82	−0.23
	p	.106	<.001	<.001	<.001	<.001	<.001	.008	<.001	.001	.194	<.001	<.001	.002	<.001		.012	<.001	<.001	<.001	<.001	<.001	<.001	<.001	<.001	<.001
%ALPHA1	r	0.04	0.01	0.01	0.02	−0.05	0.00	−0.06	0.08	0.06	0.03	0.08	0.05	−0.32	0.11	−0.05	1.00	0.91	0.09	−0.07	0.08	−0.07	−0.26	−0.29	0.11	−0.11
	p	.061	.637	.566	.414	.017	.956	.004	<.001	.003	.123	<.001	.006	<.001	<.001	.012		<.001	<.001	.001	<.001	<.001	<.001	<.001	<.001	<.001
ALPHA1	r	0.01	−0.02	−0.01	0.01	−0.05	−0.04	−0.05	0.02	−0.02	0.02	0.05	0.02	0.04	−0.09	−0.08	0.91	1.00	0.01	0.03	0.08	0.1	−0.05	−0.03	−0.09	0.00
	p	.786	.392	.515	.552	.015	.034	.007	.285	.264	.276	.021	.373	.048	<.001	<.001	<.001		.747	.159	<.001	<.001	.01	.092	<.001	.895
%ALPHA2	r	0.17	0.19	0.24	−0.06	0.19	−0.1	−0.07	−0.04	0.15	0.00	0.07	0.06	−0.22	−0.04	−0.17	0.09	0.01	1.00	0.87	−0.03	−0.13	−0.24	−0.25	−0.04	−0.09
	p	<.001	<.001	<.001	.001	<.001	<.001	.001	.043	<.001	.811	.001	.002	<.001	.037	<.001	<.001	.747		<.001	.126	<.001	<.001	<.001	.037	<.001
ALPHA2	r	0.12	0.16	0.21	−0.07	0.2	−0.15	−0.06	−0.13	0.05	−0.03	0.01	0.01	0.21	−0.29	−0.2	−0.07	0.03	0.87	1.00	−0.06	0.06	0.03	0.07	−0.29	0.05
	p	<.001	<.001	<.001	<.001	<.001	<.001	.004	<.001	.015	.106	.597	.74	<.001	<.001	<.001	.001	.159	<.001		.005	.002	.139	<.001	<.001	.016
%BETA	r	0.07	0.1	0.1	0.00	0.02	0.12	−0.05	0.00	0.05	0.06	0.02	−0.02	−0.01	−0.24	−0.26	0.08	0.08	−0.03	−0.06	1.00	0.86	−0.18	−0.15	−0.24	−0.07
	p	<.001	<.001	<.001	.814	.404	<.001	.014	.807	.009	.002	.324	.321	.626	<.001	<.001	<.001	<.001	.126	.005		<.001	<.001	<.001	<.001	<.001
BETA	r	0.02	0.06	0.07	0.00	0.03	0.07	−0.03	−0.07	−0.04	0.03	−0.03	−0.07	0.43	−0.45	−0.26	−0.07	0.1	−0.13	0.06	0.86	1.00	0.1	0.19	−0.45	0.07
	p	.216	.002	.001	.924	.116	.001	.1	<.001	.038	.16	.152	.001	<.001	<.001	<.001	<.001	<.001	<.001	.002	<.001		<.001	<.001	<.001	<.001
%GAMMA	r	−0.08	−0.06	−0.04	−0.1	0.12	−0.15	0.01	−0.5	−0.22	−0.04	0.02	−0.25	0.59	−0.77	−0.54	−0.26	−0.05	−0.24	0.03	−0.18	0.1	1.00	0.98	−0.77	0.45
	p	<.001	.001	.029	<.001	<.001	<.001	.551	<.001	<.001	.037	.213	<.001	<.001	<.001	<.001	<.001	.01	<.001	.139	<.001	<.001		<.001	<.001	<.001
GAMMA	r	−0.09	−0.06	−0.04	−0.08	0.11	−0.14	0.02	−0.45	−0.23	−0.04	−0.01	−0.23	0.71	−0.77	−0.46	−0.29	−0.03	−0.25	0.07	−0.15	0.19	0.98	1.00	−0.77	0.45
	p	<.001	.002	.033	<.001	<.001	<.001	.442	<.001	<.001	.032	.801	<.001	<.001	<.001	<.001	<.001	.092	<.001	<.001	<.001	<.001	<.001		<.001	<.001
ALB/GLOB Ratio	r	0.01	−0.04	−0.09	0.17	−0.2	0.2	0.04	0.59	0.16	0.02	−0.09	0.23	−0.56	1.00	0.82	0.11	−0.09	−0.04	−0.29	−0.24	−0.45	−0.77	−0.77	1.00	−0.36
	p	.461	.031	<.001	<.001	<.001	<.001	.065	<.001	<.001	.343	<.001	<.001	<.001	<.001	<.001	<.001	<.001	.037	<.001	<.001	<.001	<.001	<.001		<.001
ELISA	r	−0.09	−0.17	−0.16	−0.03	−0.05	−0.16	−0.05	−0.29	−0.19	0.04	0.11	−0.14	0.3	−0.36	−0.23	−0.11	0.00	−0.09	0.05	−0.07	0.07	0.45	0.45	−0.36	1.00
	p	<.001	<.001	<.001	.118	.024	<.001	.014	<.001	<.001	.031	<.001	<.001	<.001	<.001	<.001	<.001	.895	<.001	.016	<.001	<.001	<.001	<.001	<.001	

r, correlation; *p*, *p*-value.

ALAT, alanine aminotransferase (U/L); %ALB, percentage of albumin; ALB, albumin (g/dL); ALB/GLOB Ratio, albumin/globulin ratio; %ALPHA1, percentage of alpha 1 globulins; ALPHA1, absolute levels of alpha 1 globulins (g/dL); %ALPHA2, percentage of alpha 2 globulins; ALPHA2, absolute levels of alpha 2 globulins (g/dL); BASO, basophils ($10^3/\mu L$); %BETA, percentage of beta globulins; BETA, absolute levels of beta globulins (g/dL); CREA, creatinine (mg/dL); ELISA, enzyme-linked immunosorbent assay; EOS, eosinophils ($10^3/\mu L$); %GAMMA, percentage of gamma globulins; GAMMA, absolute levels of gamma globulins (g/dL); LYM, lymphocytes ($10^3/\mu L$); MON, monocytes ($10^3/\mu L$); NEU, neutrophils ($10^3/\mu L$); PCV, packed cell volume (%); PLT, platelets ($10^3/\mu L$); TP, total protein (g/dL); UREA, urea (mg/dL); WBC, white blood cells ($10^3/\mu L$).
